# Lipid profile dysregulation in opium users based on Fasa PERSIAN cohort study results

**DOI:** 10.1038/s41598-021-91533-4

**Published:** 2021-06-08

**Authors:** Maryam Kazemi, Mina Bazyar, Mohammad Mehdi Naghizadeh, Azizallah Dehghan, Massih Sedigh Rahimabadi, Mahsa Rostami Chijan, Mostafa Bijani, Maryam Zahmatkeshan, Alireza Ghaemi, Nastaran Samimi, Reza Homayounfar, Mojtaba Farjam

**Affiliations:** 1 Noncommunicable Diseases Research Center, Fasa University of Medical Sciences, Fasa, Iran; 2 Health Policy Research Center, Institute of Health, Shiraz University of Medical Sciences, Shiraz, Iran; 3 Department of Dermatology, School of Medicine, Shiraz University of Medical Sciences, Shiraz, Iran; 4 Department of Persian Medicine, Fasa University of Medical Sciences, Fasa, Iran; 5 Department of Nutrition, Health Sciences Research Center, Addiction Institute, Faculty of Public Health, Mazandaran University of Medical Sciences, Sari, Iran; 6 Student Research Committee, Fasa University of Medical Sciences, Fasa, Iran; 7 National Nutrition and Food Technology Research Institute (WHO Collaborating Center), Faculty of Nutrition Sciences and Food Technology, Shahid Beheshti University of Medical Sciences, Tehran, Iran; 8 Clinical Research Development Unit, Vali-E Asr Hospital, Fasa University of Medical Sciences, Fasa, Iran

**Keywords:** Cardiology, Diseases, Health care, Medical research, Risk factors

## Abstract

One of the main health problems in many societies is the increased opium abuse, which was found to be correlated with many problems like cardiovascular disease. This study aimed to evaluate the correlation of opium use with blood lipoproteins as the risk factor of CVD. This was a cross-sectional study conducted on participants of the first phase of the PERSIAN Cohort study who were aged between 35 and 70 years old. Demographic characteristics; history of smoking, alcohol, and opium consumption; medical history; and medications were asked and the related checklists were filled out. The levels of physical activity and fat intake were also registered. As well, lipoprotein profiles were investigated by blood sampling. The linear and logistic regression was used to analyze the relationship between opium and lipid profile and the statistical significant level was considered as < 0.05. Among 9300 participants with a mean age of 48.06 ± 9.44 years old, 49.6% of them were men. About 24.1% of the participants used opium. In the linear regression models, unlike TG (β = 2.2, p = 0.36), total cholesterol (β = − 2.5, p = 0.02), LDL (β = − 2.0, p = 0.04), and HDL (β = − 1.0, p = 0.04) were significantly lower in people who used opium compared to the others. In the logistic regression models, abnormal level of LDL (OR = 0.78, p = 0.003) and total cholesterol (OR = 0.82, p = 0.008) were less in people who used opium compared to the others. This study showed that there is a correlation between opium usage and lower levels of total cholesterol and LDL; however, the lower level of HDL in normal range was seen in opium users. Considering the current evidences, most of them showed the increased risks of ischemic heart disease, heart attack, hypertension, cerebrovascular disease, and cancer in opium users. Therefore, Healthcare providers and patients should be noticed about the deleterious effects of opium consumption on various vascular events. In addition, it is necessary for managers and policy makers of the health care system to take the necessary measures to raise the level of awareness and health literacy of the general public about the high-risk side effects of opium use and to take necessary and effective strategies to prevent and reduce its use.

## Introduction

According to World Drug Report, about 58 million adult people use one type of opium worldwide in 2019^1^. Evidence showed that opium is traditionally used in many south and central Asian countries, including India, Iran, Afghanistan, and Pakistan^2^. In these cultures, people believe that opium usage is effective on controlling blood sugar, blood pressure, and lipids that is one of the causes of the increased prevalence of opium usage among middle age and elderly^3^. In total, the prevalence of drug abuse is globally increasing unfortunately, and addiction is going to become one of the current important issues. Prevalence of opium usage in Iran is vary from 8.9% in rural area of Babol^4^ to 17% in Golestan^5^ and 24.7% in rural area of Kerman^6^.

Cardiovascular, respiratory, and Central Nervous Systems are mostly affected by opium abuse. Most studies showed that opium usage increases the risks of acute myocardial infarction, atherosclerosis, and cardiovascular mortality^7^. Additionally, opium usage increases the risk of many cancers such as lung, esophageal, gastric, laryngeal, and bladder cancers^2^.

Lipid profiles are known as the risk factors of cardiovascular disease and many studies assessed the correlation between opium abuse and lipid level. Aghadavoudi et al. in their study showed that LDL and triglyceride levels were higher in drug addicts than non-drug-dependent groups^8^. According to World Health Organization reports, 50% of mortality in developed and 30% of mortality in developing countries are due to coronary artery disease, one of the important and common factors of which is opium abuse^9,10^. However, there are many controversies about opium effect on lipid profile. In a study by Fatemi et al., it was confirmed that cholesterol reduces in people consuming opium compared with people who do not consume it^11^. While in other studies, it was pointed out that the levels of triglyceride, total cholesterol, and LDL significantly increased in the drug addict group, but HDL levels did not change significantly^12^. To investigate the correlation of opium with lipid profile, there are many confounding factors that could affect it such as smoking and alcohol consumption. So, in this study, the correlation between opium and lipid profile was evaluated considering these confounding factors in a remarkable population with sufficient sample size consisting of those who used opium in the Fasa cohort study.

## Methods and materials

### Study design and participants

This study was a cross-sectional study performed as the first phase of a longitudinal Fasa branch of PERSIAN (prospective Epidemiological Research Study in Iran) cohort study on the population of Fasa, the south of Iran. Geographically, Fasa is a city in the southeast of Fars province with total population of about 250,000 people. Correspondingly, one of its districts is called Sheshdeh (28° 56′ 56.0″ N 53° 59′ 26.9″ E), which contains 41,000 people who were chosen for the Fasa cohort study. The subjects in the age range of 35–70 years old were invited to participate in this study who were about 10,000 people. The protocol of this population-based mega project was presented by Malekzadeh and Farjam et al. in 2016. Biological sample collection of basic data was defined in the cohort study’s protocol^13–15^.

**Figure 1:**
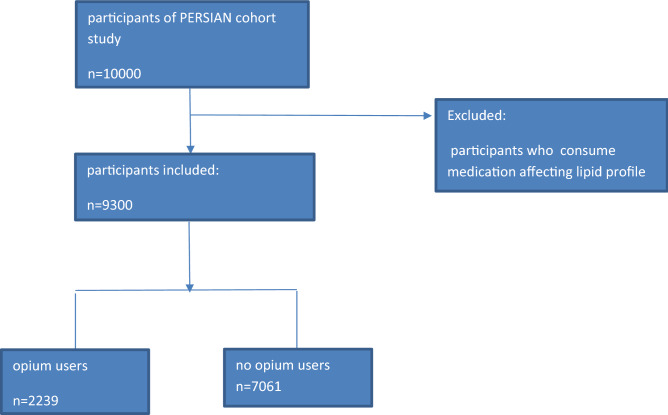
Flowchart of the study design of lipid profile in opium users.

Those people who were consuming medication affecting lipid profile such as additive containing Alcohol-Isotretinoin (Accutane, Roaccutane), OCP (Estrogen, Progesterone), Methylprednisolone, Prednisolone, Betamethasone, Dexamethasone, Hydrocortisone, HDL additive and TG, Chol, and LDL reducer (Atorvastatin, Lovastatin, Simvastatin, Fluvastatin, Gemfibrozil, Clofibrate, Fenofibrate, Niacin, Ezetimibe, Cholestyramine) were excluded from the total participants of the cohort, and finally 9300 people were included in our study (Fig. 1).

### Study instruments and variable’s assessment

Each participant was interviewed based on a questionnaire approved by the PERSIAN cohort consortium in Islamic republic of Iran. Data gathering tools were the general information questionnaire (sex, age, marital status, education level, job records, socioeconomic status, and place of living) and clinical information questionnaire (history of chronic diseases and the used medications). All the individuals who entered the study were evaluated for weight, height, and BMI. Blood sampling was done for all the participants to check lipid Profiles, including Low Density Lipoprotein (LDL), High Density Lipoprotein (HDL), Triglyceride (TG), and Cholesterol (colorimetry, pars Azmoon kit). Abnormal levels of TG, Chol, HDL, and LDL were considered as 150 mg/dl, 200 mg/dl, and 40 mg/dl for men and 50 mg/dl and 130 for women, respectively (https://labtestsonline.org/test/lipid-panel).

History of smoking, opium, and alcohol usage was recorded. The participants were asked if they are active current smoker, or use opium derived products or alcohol frequently during a week.

The Validated Food Frequency Questionnaire (FFQ) and International Physical Activity Questionnaire (IPAQ) were filled out to evaluate fat intake (Kcal) and Physical activity (Met-min/week) in the participants of this study.

There are many confounding factors such as age, gender, BMI, Fat intake, medications, smoking, and alcohol consumption that were used as confounders in the regression model.

### Statistical analysis

The lipid profile in the quantitative format was compared between socio-demographic characteristics via t-test, ANOVA, and correlation coefficient. The multivariable linear regression model was also used to adjust and remove confounding factors’ effects from the relationship between opium using and the lipid profiles. In this model, the P-value and beta coefficient with its standard error of opium using variable were reported. Moreover, the lipid profile in the qualitative format was compared between socio-demographic characteristics via the chi-square test. The multivariable logistic regression model was used to adjust and remove confounding factors’ effects from the relationship between opium using and the levels of lipid profiles. In both of the above-mentioned models, variables that had a p-value less than 0.2 in the univariate analysis were considered as candidates for entering the model as the predictors. Because our aim of regression analysis was eliminating confounding effect of predictors, all selected predictors were forced to entering in the linear and logistic regression models (the stepwise variable selection was not used). IBM SPSS Statistics v21 was used and a P-value < 0.005 was considered as the significance level.

### Ethics approval and consent to participate

The study protocol was in accordance with the Helsinki Declaration. The study protocol was approved by National and Regional Ethics Committee of FUMS (code: IR.FUMS.REC.1396.234) and Research Board of Fasa University of medical Sciences (code: 94153). A written and informed consent was obtained from each participant to enter the first phase of cohort study.

### Approval code

IR.FUMS.REC.1396.234.

## Results

This study was conducted on 9300 participants with the mean age of 48.06 ± 9.44 years old. Notably, 4340 (46.7%) cases were men. Their mean years of education was 4.8 ± 3.89. Of total, 8318(89.4%) subjects were married and 4867(52.3%) subjects had job (Table 1).

**Table 1:** Socio demographic characteristics of the opium and non-opium users in first phase of PERSIAN cohort study.

Variable	Opium user n = 2239 (24.1%)		Opium non-user n = 7061 (75.9%)		Total	
	n	%	n	%	n	%
**Gender**						
Male	2152	49.6	2188	50.4	4340	46.7
Female	87	1.89	4873	98.2	4960	53.3
**Job status**						
Employed	1946	40	2921	60	4867	52.3
Unemployed	291	6.6	4125	93.4	4416	47.5
**Marital status**						
Single	53	14.8	304	85.2	357	3.8
Married	2163	26	6155	74	8318	89.4
widow	12	2.3	517	97.7	529	5.7
Divorced	11	11.5	85	88.5	96	1
**Socio-economic status**						
Low	656	21.4	2411	78.6	3067	33
Middle	722	24.2	2262	75.8	2984	32.1
High	843	26.6	2322	73.4	3165	34

Variable	Mean	SD	Mean	SD	Mean	SD
Age (year)	47.13	8.58	48.36	9.68	48.06	9.44
Education (years)	5.78	3.80	4.48	3.87	4.80	3.89
BMI (kg/m^2^)	23.39	4.54	26.22	4.72	25.54	4.83
Physical activity (Met-h/day)	44.90	14.38	40.74	10.21	41.74	11.49
Fat intake (g/day)	89.78	42.35	72.69	34.91	78.8	37.55
TG	130.0	81.0	133.6	87.8	130.9	82.7
CHOL	189.4	38.6	176.8	37.0	186.4	38.6
HDL.C	52.4	16.2	46.9	14.4	51.1	16.0
LDL	111.0	32.4	103.2	31.0	109.1	32.2

2564 (27.6%) people were current smoker, 2239 (24.1%) were opium users and 196 (2.1%) consumed alcohol regularly.

In this population Mean of TG was 130.88 ± 82.72, Cholesterol was 186.38 ± 38.58, LDL was 109.1 ± 32.22, and HDL was 51.06 ± 15.98 mg/dl.

Univariate analysis show that age (r = 0.15, p < 0.001), years of education (r = − 0.11, p < 0.001), BMI (r = 0.20, p < 0.001), physical activity base on MET (r = − 0.09, p < 0.001), fat intake (r = − 0.05, p < 0.001) were significantly correlated with cholesterol level. Female (p < 0.001), unemployed (p < 0.001), and widow participants (p < 0.001) had significantly higher level of cholesterol.

Triglyceride level was not correlated with Age (r = 0.01, p = 0.07), years of education (r = − 0.0.01, p = 0.19) and fat intake (r = -0.009, p = 0.36) but, BMI (r = 0.21, p < 0.001) and physical activity base on MET (r = − 0.05, p < 0.001) were significantly correlate with TG. Female (p < 0.001), single and divorced (p < 0.001), low socioeconomic level (p < 0.001) participants had significantly lower level of TG.

Lower level of HDL was seen in male (p < 0.001), married (p < 0.001), employed (p < 0.001) and high socio-economic status (p < 0.001) participants. Age (r = 0.08, p < 0.001), years of education (r = − 0.13, p < 0.001) and BMI (r = − 0.02, p = 0.01) were significantly correlated but physical activity (r = − 0.01, p = 0.3) and fat intake (r = 0.005, p = 0.65) were not correlated with HDL level.

Age (r = 0.13, p < 0.001), years of education (r = − 0.07, p < 0.001), BMI (r = 0.14, p < 0.001), physical activity (r = − 0.08, p < 0.001) and fat intake (r = − 0.05, p < 0.001) were significantly correlated with LDL level. Female (p < 0.001), widow (p < 0.001) and unemployed (p < 0.001) persons had higher level of LDL (“ESM Appendix”).

**Figure 2:**
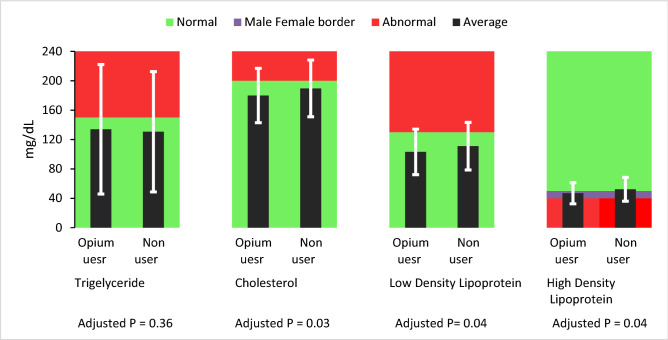
Comparison of lipid profile between opium user and non-user at the first of phase of PERSIAN cohort study. Lipid profile is presented with the mean—black bars—and standard deviation—white error bars—in mg/dL. The normal and abnormal ranges of lipid profiles are colored in green and red background. The border of the normal and abnormal HDL between the male and female subjects was also colored as purple background. The p-values came from a linear multivariable model that all variables having a correlation with lipid profile in univariate analysis with a p-value less than 0.2, were added to it. The p-value of the opium use variable in this model was reported as the Adjusted p-value.

**Figure 3:**
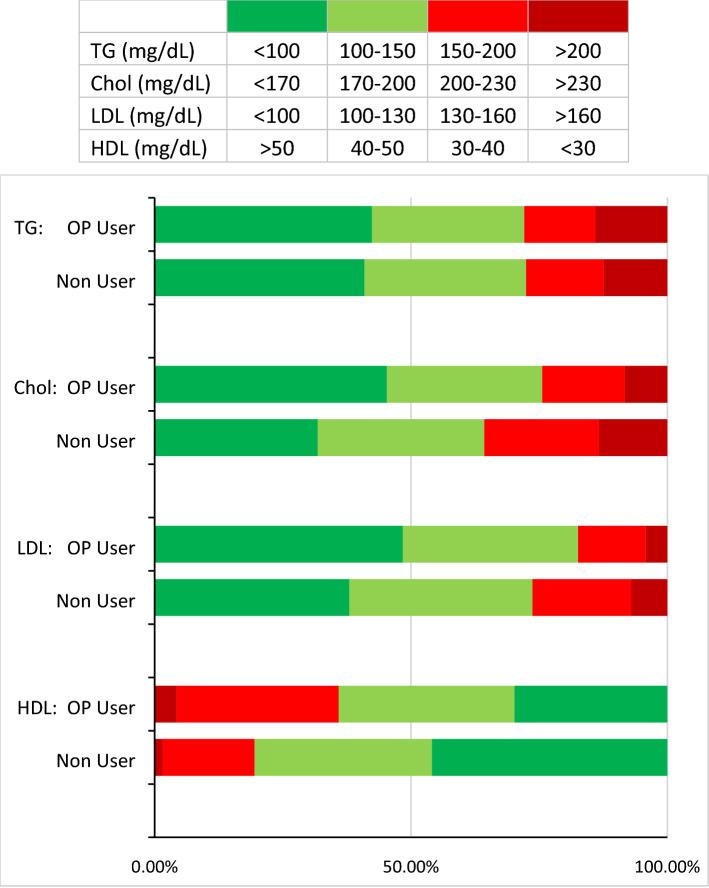
Percentage of participants with normal and abnormal levels of lipid profile in both user and non-user groups of opium. The normal range was presented as green (dark green as healthy and light green as normal borderline) and the abnormal range was presented as red color (light red as abnormal borderline and dark red as unhealthy). The levels of TG, cholesterol, LDL, and HDL were presented in the above table.

In a linear regression model, we added all variables which were correlate with lipid profile (Chol, TG, LDL, HDL) in univariate analysis with significant level of < 0.2. Adjusted p value was reported for correlation of Opium and lipid profile. Opium users had significantly lower level of total Cholesterol (β = − 2.55, SE-β = 1.20, p = 0.034), LDL (β = − 2.03, SE-β = 1.02, p = 0.045) and HDL (β = − 1.05, SE-β = 0.497, p = 0.036), but TG had not significantly relation with using opium (β = − 2.17, SE-β = 2.41, p = 0.359) (Figs. 2, 3).

Due to the importance of high lipid profile as risk factor for many diseases such as cardio vascular, we analyzed correlation of opium usage and abnormal level of lipid profile in present of other variable and confounders in logistics regression model (Table 2).

**Table 2:** logistic regression model for lipid profile in first phase of PERSIAN cohort Study.

	TG > 150			Chol > 200			LDL > 130				HDL < 40 male < 50 female		
	p-value	OR	95% CI for OR	p-value	OR	95% CI for OR	p-value	OR	95% CI for OR	p-value		OR	95% CI for OR
Opium use	0.646	1.032	0.902–1.180	0.008	0.822	0.710–0.951	0.003	0.785	0.667–0.923	0.410		0.830	0.532–1.293
Marital state (married)	0.208	1.199	0.904–1.591	0.793	1.035	0.803–1.333	0.133	1.247	0.935–1.663	0.022		0.734	0.564–0.956
Marital state (widow)	0.013	1.548	1.097–2.184	0.458	1.125	0.824–1.535	0.021	1.500	1.064–2.114	0.001		0.568	0.413–0.782
Marital state (divorced)	0.994	1.002	0.561–1.792	0.910	0.971	0.582–1.621	0.442	1.242	0.715–2.155	0.838		0.948	0.569–1.580
Socioeconomic (middle)	0.314	1.063	0.943–1.198	0.123	1.093	0.976–1.224	NUiM			0.309		1.075	0.935–1.237
Socioeconomic (high)	0.566	1.038	0.915–1.176	0.610	1.032	0.915–1.164	NUiM			0.574		1.045	0.897–1.217
Job (unemployed)	0.127	1.115	0.970–1.281	0.268	1.076	0.945–1.226	0.048	1.153	1.001–1.329	0.002		0.799	0.692–0.923
Smoking	NUiM			0.525	0.957	0.836–1.095	0.232	0.913	0.787–1.060	0.574		0.923	0.700–1.219
Alcohol consuming	0.046	1.385	1.006–1.909	0.001	1.746	1.258–2.424	0.002	1.774	1.241–2.536	< 0.001		1.020	1.012–1.029
Gender (Female)	< 0.001	0.655	0.563–0.762	< 0.001	1.342	1.157–1.557	0.001	1.315	1.120–1.545	0.368		0.990	0.967–1.012
Age (years)	0.090	1.005	0.999–1.012	< 0.001	1.035	1.029–1.042	< 0.001	1.034	1.027–1.040	< 0.001		0.958	0.947–0.970
Education years (years)	0.966	1.000	0.984–1.017	0.989	1.000	0.984–1.016	0.004	1.025	1.008–1.042	NUiM			
BMI (kg/m^2^)	< 0.001	1.114	1.103–1.126	< 0.001	1.063	1.053–1.074	< 0.001	1.046	1.035–1.057	NUiM			
Physical activity (Met-h/day)	< 0.001	0.990	0.985–0.995	0.060	0.995	0.991–1.000	0.010	0.993	0.988–0.998	NUiM			
Fat intake (g/day)	NUiM			0.327	1.001	0.999–1.002	0.266	0.999	0.998–1.001	NUiM			

## Discussion

This study was conducted on the rural population of the Fasa PERSIAN cohort study with a mean age of 48 years old. This study was conducted on 9300 participants with the mean age of 48.06 ± 9.44 years old. About 24.1% of the participants used opium. In this population Mean of TG was 130.88 ± 82.72, Cholesterol was 186.38 ± 38.58, LDL was 109.1 ± 32.22, and HDL was 51.06 ± 15.98 mg/dl. Univariate analysis show that age (r = 0.15, p < 0.001), years of education (r = − 0.11, p < 0.001), BMI (r = 0.20, p < 0.001), physical activity base on MET (r = − 0.09, p < 0.001), fat intake (r = − 0.05, p < 0.001) were significantly correlated with cholesterol level. Female (p < 0.001), unemployed (p < 0.001), and widow participants (p < 0.001) had significantly higher level of cholesterol. Triglyceride level was not correlated with Age (r = 0.01, p = 0.07), years of education (r = − 0.0.01, p = 0.19) and fat intake (r = − 0.009, p = 0.36) but, BMI (r = 0.21, p < 0.001) and physical activity base on MET (r = − 0.05, p < 0.001) were significantly correlate with TG. Female (p < 0.001), single and divorced (p < 0.001), low socioeconomic level (p < 0.001) participants had significantly lower level of TG.

In the linear regression models, unlike TG (β = 2.2, p = 0.36), total cholesterol (β = − 2.5, p = 0.02), LDL (β = − 2.0, p = 0.04), and HDL (β = − 1.0, p = 0.04) were significantly lower in people who used opium compared to the others. In the logistic regression models, abnormal level of LDL (OR = 0.78, p = 0.003) and total cholesterol (OR = 0.82, p = 0.008) were less in people who used opium compared to the others.

The prevalence of opium usage was 24 people per 100 individuals. The frequency of the current cigarette smokers and regular alcohol consumption was 27% and 2%, respectively. One of the considerable causes for the high prevalence of opium abuse in this study compare to the other studies^4,5,16^ was found to be the location of Fasa city in the route of transportation of opium. Mainly, the route of transportation of drugs into the country from the eastern border (Kerman province) and from the southern borders (Hormuzgan province) crosses the city of Fasa. So, people have more access to this kind of substance^17^.

Due to living in Middle Eastern societies, people believe that consumption of opium is effective on controlling blood pressure, lipids, and glucose and on preventing heart diseases^18^. As well, there are many studies with different results performed on the opium effects on lipid profile. Some studies have suggested that opium use has no significant effect on total cholesterol, LDL or HDL-C^19^. While some other studies pointed out that the levels of triglyceride, total cholesterol, and LDL significantly increase in the drug addict groups, but HDL levels did not change significantly in the studies rabbits^12^. In a systematic review and meta-analysis study on diabetic patients, the results showed that total cholesterol was lower in opium abusers, but no significant changes were observed in other lipid profile between users and non-users^20^. Furthermore, our findings demonstrated that opium users have lower levels of total cholesterol, LDL, and HDL in comparison with non-users. But opium usage had no correlation with the TG level.

The relationship between low cholesterol level and opioid signaling has been previously studied^21^. Lipid rafts microdomains exist in the outer layer of the plasma membrane, which contain high levels of cholesterol. Additionally, these microdomains host opioid receptors, including µ-opioid receptor (MOR), κ opioid receptors (KOR), and δ-opioid receptors (DOR) as well as various signaling factors like G protein-coupled receptors (GPCR)^22,23^. The process of opioid signaling has many steps, including desensitization, phosphorylation, internalization, and re-sensitization. Internalization is considered as the primary step leading to the re-sensitization of the opioid receptors^24^. It has been reported that cholesterol depletion could reduce the internalization of δ-opioid receptors in HEK293 cells^25^. According to a study by Zheng et al., reducing the cholesterol level by simvastatin disrupted the opioid signaling in the cultured neurons and decreased the analgesic effect of opioids in a mouse model^21^. Moreover, a clinical study conducted by Huang et al. indicated that patients with low levels of cholesterol may require higher doses of opioids, in order to reduce their pain^26^. Altogether, it seems that cholesterol plays an important role in the opioid signaling.

Our result show significantly lower levels of cholesterol among opium users. In addition, we mentioned some previous studies emphasizing on that low cholesterol levels impair the opioid signaling. However, the chronology of low cholesterol levels and opioid tolerance among amusers is not clear yet. Therefore, more experimental and clinical studies should be conducted to determine if opium abuse causes low cholesterol levels or if low cholesterol level accelerates the process of tolerance in opioid abusers. It is known that opioid abuse dramatically changes the diet and consequently causes the loss of appetite and malnutrition in most cases^27^. It is possible that lack of proper diet leads to the reduced cholesterol levels in the opium abusers that cause more opioid tolerance, so these individuals consequently require the increased opium dose over time. However, this hypothesis requires more precise studies to be proven.

Although lower levels of cholesterol in opium abusers could be considered due to the decreased appetite and nutritional deficiency in them^28,29^, it was shown that opiate agonists by the effect of κ receptors could cause the decreased carbohydrate to fat ratio intake in rats^30^. However, in our study, by including fat intake, BMI, and other confounders, a lower level of total cholesterol was seen in opium users.

The importance of serum lipid profile is known as a risk factor of cardiovascular diseases. Furthermore, lipids profile is one of the most important risks of metabolic syndrome and 10 years risk of cardiovascular disease. So, it is necessary to pay attention to the correlation between opium consumption and serum lipids due to the cut points that are known as hyperlipidemia in the clinic. Considering the cut point of abnormal level for serum lipids by including other variables and confounders in the correlation between opium and lipid profile, we found that opium usage was protective for increasing the levels of total cholesterol and LDL to an abnormal cut point, but not for TG and HDL levels. Marmor M et al. showed that the usage of opium or morphine could have a protective effect against cardiovascular diseases^18^, which is aligned with our results. While in contrast, many other studies showed that opium addiction is correlated with the increased risk of cardiovascular diseases^31–35^, and usage of opium increases the risk of death with several causes like circulatory diseases^36^. Contradiction in these results could be due to the variations in method, dosage, and duration of consumption of opium in different studies.

Possibly, by performing more studies, the level of opium consumption due to its effect on lipid profile would be defined as what has been done for harmful alcohol dosage.

According to Ziaee et al., study showed that although opium consumption causes a relative decrease in LDL and cholesterol levels in patients, increasing the amount and timing of these compounds in the long-term can consequently cause changes in plasma fibrinogen levels, coagulation, and atherosclerosis and also exacerbate coronary artery diseases (CAD), hypertension, and stroke^37^. The data obtained from a large prospective cohort study showed that the increased risk of death from circulatory diseases is associated with opium consumption. Unfortunately, misconceptions about the positive effects of opium are widespread, and healthy people as well as patients with coronary artery diseases or diabetes, should be informed of the dangerous effects of opium consumption on the increased risk of cardiometabolic diseases. Furthermore, it is recommended to promote the knowledge of medical communities and staff on the potential health consequences of opium consumption^10^. In this regard, the results of a study conducted in Iran showed that 15% of hospitalized patients with myocardial infarction and coronary heart disease were dependent on opium consumption^38^. The results of a study by Khalili et al.^32^ showed that the use of opium compounds causes a relative decrease in both LDL and cholesterol levels. However, a significant relationship was reported among the chances of ischemic heart disease, heart attack, blood pressure, and opium use. According to the results of this study, 46.19% of male and 4.27% of female subjects have used opium compounds at least once in past six months. Additionally, a significant relationship was observed among opium use and education level, economic and social level, smoking, and alcohol consumption, which is in line with the results of the present study. Therefore, it is necessary for health system policymakers to use the necessary plans, in order to increase public awareness on opium consumption^9^.

The results of a study by Sheikh et al.^39^ on 50045 people aged between 45 and 70 years old based on the data obtained from the Golestan Cohort in northern Iran showed that there were some significant positive correlations among the opium consumption and gastrointestinal cancers, esophageal cancer, lung cancer, bladder cancer, pancreatic cancer, and liver cancer. Hence, some strategies should be developed and implemented for the prevention and cessation of opioid abuse^39^.

People are using opium for many years, not only as a habit, but also based on their traditional beliefs about its beneficial effects on diabetes mellitus, dyslipidemia, and cardiovascular disorder. Considering the current evidences, opium not only has no protective effect on cardiac diseases, but the clinical and prospective cohort studies also consistently indicated that opium consumption is associated with cardiovascular diseases and cardiovascular mortality. Unfortunately, false beliefs on the beneficial effects of opium are common, and it is the responsibility of health professionals to battle against these false beliefs^7^.

## Strengths of the study

As the strength of our study, we can mention the elimination of many confounding factors, including medication, fat intake, BMI, and physical activity. This study was a population-based research performed with a large sample of people. Additionally, we used one type of device as well as a single laboratory to measure the amount of blood lipids in all the included participants.

## Limitation

Given the fact that the opium consumption is influenced by cultural, social, and economic factors, so the results of this study cannot be generalized to other parts of Iran and other countries and it is necessary to conduct similar studies in other parts of Iran and also in other countries. As well, in this study, the outcomes related to the side effects of opium consumption with cardiovascular disease, hypertension, cerebrovascular diseases, and cancer have not been investigated, which are suggested to be studied in future studies. In the present study, those people who were consuming medication affecting lipid profile such as additive containing Alcohol-Isotretinoin (Accutane, Roaccutane), OCP (Estrogen, Progesterone), Methylprednisolone, Prednisolone, Betamethasone, Dexamethasone, Hydrocortisone, HDL additive and TG, Chol, and LDL reducer (Atorvastatin, Lovastatin, Simvastatin, Fluvastatin, Gemfibrozil, Clofibrate, Fenofibrate, Niacin, Ezetimibe, Cholestyramine) were excluded and confounding factors such as diet regime, physical activity, smoking and alcohol consumption, have entered the study. As well as temporality and potential reverse causation it has not been investigated. Accordingly, it is suggested that this relationship be considered in the follow-up phase of this prospective study.

## Conclusion

Lower levels of total cholesterol and HDL-C were seen in opium users, but by considering abnormal cut point for lipid profiles, opium users had normal levels of LDL and total cholesterol in comparison with non-users who had abnormal levels. Despite our results, opium usage is not recommended for decreasing lipid profile and risk of heart disease, because opium has some known side effects on many organs and causes other non-communicable diseases like cancers. Considering the current evidences, most of them showed the increased risks of ischemic heart disease, heart attack, hypertension, cerebrovascular disease, and cancer in opium users. Therefore, Healthcare providers and patients should be noticed about the deleterious effects of opium consumption on various vascular events. In addition, it is necessary for managers and policy makers of the health care system to take the necessary measures to raise the level of awareness and health literacy of the general public about the high-risk side effects of opium use and to take necessary and effective strategies to prevent and reduce its use.

## Supplementary Information

Supplementary Information.

## Data Availability

The datasets were analyzed during the study are available from the corresponding author on reasonable request.
