# Publisher Correction: Lipid profile dysregulation in opium users based on Fasa PERSIAN cohort study results

**DOI:** 10.1038/s41598-022-08883-w

**Published:** 2022-03-24

**Authors:** Maryam Kazemi, Mina Bazyar, Mohammad Mehdi Naghizadeh, Azizallah Dehghan, Massih Sedigh Rahimabadi, Mahsa Rostami Chijan, Mostafa Bijani, Maryam Zahmatkeshan, Alireza Ghaemi, Nastaran Samimi, Reza Homayounfar, Mojtaba Farjam

**Affiliations:** 1 Noncommunicable Diseases Research Center, Fasa University of Medical Sciences, Fasa, Iran; 2 Health Policy Research Center, Institute of Health, Shiraz University of Medical Sciences, Shiraz, Iran; 3 Department of Dermatology, School of Medicine, Shiraz University of Medical Sciences, Shiraz, Iran; 4 Department of Persian Medicine, Fasa University of Medical Sciences, Fasa, Iran; 5 Department of Nutrition, Health Sciences Research Center, Addiction Institute, Faculty of Public Health, Mazandaran University of Medical Sciences, Sari, Iran; 6 Student Research Committee, Fasa University of Medical Sciences, Fasa, Iran; 7 National Nutrition and Food Technology Research Institute (WHO Collaborating Center), Faculty of Nutrition Sciences and Food Technology, Shahid Beheshti University of Medical Sciences, Tehran, Iran; 8 Clinical Research Development Unit, Vali-E Asr Hospital, Fasa University of Medical Sciences, Fasa, Iran

Correction to: *Scientific Reports* https://doi.org/10.1038/s41598-021-91533-4, published online 08 June 2021

The original version of this Article contained errors.

Firstly, six References were omitted. These References are now cited at the relevant points in-text and are listed below:

1. United Nations. World Drug Report 2020. Available at https://wdr.unodc.org/wdr2020/en/exsum.html (2020).

21. Zheng, H. *et al.* Cholesterol level influences opioid signaling in cell models and analgesia in mice and humans. *J. Lipid Res.* **53**(6), 1153–1162 (2012).

24. Nagi, K. & Piñeyro, G. Regulation of opioid receptor signalling: implications for the development of analgesic tolerance. *Mol. Brain.* **4**(1), 1–9 (2011).

25. Brejchova, J. *et al.* Plasma membrane cholesterol level and agonist-induced internalization of δ-opioid receptors; colocalization study with intracellular membrane markers of Rab family. *J. Bioenerg. Biomembr.* **48**(4), 375–396 (2016).

26. Huang, Z. *et al.* Opioid doses required for pain management in lung cancer patients with different cholesterol levels: negative correlation between opioid doses and cholesterol levels. *Lipids Health Dis.* **15**(1), 1–9 (2016).

27. Santolaria-Fernàndez, F. J. *et al.* Nutritional assessment of drug addicts. *Drug Alcohol Depend.* **38**(1), 11–18 (1995).

As a result, the following changes were made to existing citations in the Discussion section.

“The relationship between low cholesterol level and opioid signaling has been previously studied^3^.”

now reads:

“The relationship between low cholesterol level and opioid signaling has been previously studied^21^.”

“Internalization is considered as the primary step leading to the re-sensitization of the opioid receptors^3^. It has been reported that cholesterol depletion could reduce the internalization of δ-opioid receptors in HEK293 cells^5^.”

now reads:

“Internalization is considered as the primary step leading to the re-sensitization of the opioid receptors^24^. It has been reported that cholesterol depletion could reduce the internalization of δ-opioid receptors in HEK293 cells^25^”

“According to a study by Zheng et al., reducing the cholesterol level by simvastatin disrupted the opioid signaling in the cultured neurons and decreased the analgesic effect of opioids in a mouse model. Moreover, a clinical study conducted by Huang et al. indicated that patients with low levels of cholesterol may require higher doses of opioids, in order to reduce their pain^4^.”

now reads:

“According to a study by Zheng et al., reducing the cholesterol level by simvastatin disrupted the opioid signaling in the cultured neurons and decreased the analgesic effect of opioids in a mouse model^21^. Moreover, a clinical study conducted by Huang et al. indicated that patients with low levels of cholesterol may require higher doses of opioids, in order to reduce their pain^26^.”

“It is known that opioid abuse dramatically changes the diet and consequently causes the loss of appetite and malnutrition in most cases^2^.”

now reads:

“It is known that opioid abuse dramatically changes the diet and consequently causes the loss of appetite and malnutrition in most cases^27^.

Due to the above, and partially due to miscommunication between the Authors, the existing References and citations also contained further errors.

References 2-8 were incorrectly listed as References 1-7.

Reference 9 was incorrectly listed as Reference 32.

Reference 10 was incorrectly listed as Reference 30.

References 11-20 were incorrectly listed as References 8-17.

References 22 and 23 were incorrectly listed as References 18 and 19.

References 28-37 were incorrectly listed as References 20-29.

Reference 38 was incorrectly listed as Reference 31.

Reference 39 was incorrectly listed as Reference 33.

In the Introduction section, citations to References 9-13 were inaccurate by 2.

Also in the Introduction section,

“According to World Health Organization reports, 50% of mortality in developed and 30% of mortality in developing countries are due to coronary artery disease, one of the important and common factors of which is opium abuse^8^”

now reads:

“According to World Health Organization reports, 50% of mortality in developed and 30% of mortality in developing countries are due to coronary artery disease, one of the important and common factors of which is opium abuse^9-10^”

“In a study by Fatemi et al., it was confirmed that cholesterol, triglyceride, and LDL-c significantly reduce in people consuming opium compared with people who do not consume it^9^.”

now reads:

“In a study by Fatemi et al., it was confirmed that cholesterol reduces in people consuming opium compared with people who do not consume it^11^.”

In the Discussion section, the sentence “In contrast, a study by Fatemi et al. confirmed that LDL significantly reduces in people consuming opium^8^.” has been removed.

Also in the Discussion section,

“While some other studies pointed out that the levels of triglyceride, total cholesterol, and LDL significantly increase in the drug addict people, but HDL levels did not change significantly in the studies rabbits^9^.”

now reads:

“While some other studies pointed out that the levels of triglyceride, total cholesterol, and LDL significantly increase in the drug addict groups, but HDL levels did not change significantly in the studies rabbits^12^.”

“One of the considerable causes for the high prevalence of opium abuse in this study compare to the other studies^3,4,13^ was found to be the location of Fasa city in the route of transportation of opium.”

now reads:

“One of the considerable causes for the high prevalence of opium abuse in this study compare to the other studies^4,5,16^ was found to be the location of Fasa city in the route of transportation of opium.”

“Unfortunately, false beliefs on the beneficial effects of opium are common, and it is the responsibility of health professionals to battle against these false beliefs^34^.”

now reads:

“Unfortunately, false beliefs on the beneficial effects of opium are common, and it is the responsibility of health professionals to battle against these false beliefs^7^.”

The original Article has been corrected.

