# Supplementary material for: Lipid profile dysregulation in opium users based on Fasa PERSIAN cohort study results
Source: Sci Rep. 2021 Jun 8;11:12058. doi: 10.1038/s41598-021-91533-4 (PMC8187592; doi:10.1038/s41598-021-91533-4)
Supplement: Supplementary file 1 — Supplementary Information. [file 41598_2021_91533_MOESM1_ESM.docx]

**Lipid Profile in Opium Users: Results of Fasa PERSIAN Cohort Study, First Phase**

Maryam Kazemi^1,2^, Mina Bazyar^3^, Mohammad Mehdi Naghizadeh^1^, Azizallah Dehghan^1^, Massih Sedigh Rahimabadi^1,4^, Mahsa Rostami Chijan^1,4^, Mostafa Bijani^1^, Maryam Zahmatkeshan^1^, Alireza Ghaemi^5^, Nastaran Samimi^6^, Reza Homayounfar^1,7^*, Mojtaba Farjam^1^*

1. Noncommunicable Diseases Research Center, Fasa university of Medical Sciences, Fasa, Iran

2. Health Policy Research Center, Institute of Health, Shiraz University of Medical Sciences, Shiraz, Iran

3. Department of Dermatology, School of Medicine, Shiraz University of Medical Sciences, Shiraz, Iran

4. Department of Persian Medicine, Fasa University of Medical Sciences, Fasa. Iran.

5. Department of Nutrition, Health Sciences Research Center, Addiction Institute, Faculty of Public Health, Mazandaran University of Medical Sciences, Sari, Iran

6. Student Research Committee, Fasa University of Medical Sciences, Fasa, Iran

7. National Nutrition and Food Technology Research Institute (WHO Collaborating Center), Faculty of Nutrition Sciences and Food Technology, Shahid Beheshti University of Medical Sciences, Tehran, Iran

Appendix1 (Table 2): Correlation of lipid profile and socio-demographic characteristics of participants in first phase of PERSIAN cohort study

| variable | Cholesterol  mean±SD | P value | TG  mean±SD | P value | LDL  mean±SD | P value | HDL  mean±SD | P value |
| --- | --- | --- | --- | --- | --- | --- | --- | --- |
| **Gender** |  |  |  |  |  |  |  |  |
| Male | 179.82±37.22 | <0.001 | 136.17±92.23 | <0.001 | 105.26±31.06 | <0.001 | 47.28±14.39 | <0.001 |
| Female | 192.3±38.85 |  | 127.2±74.65 |  | 112.47±32.86 |  | 54.36±16.56 |  |
| **Job** |  |  |  |  |  |  |  |  |
| Employed | 181.61±37.9 | <0.001 | 142.53±87.03 | 0.16 | 106.56±31.47 | <0.001 | 48.49±14.64 | <0.001 |
| Unemployed | 191.84±38.68 |  | 130.14±79.31 |  | 111.9±32.84 |  | 53.87±16.88 |  |
| **Marital status** |  |  |  |  |  |  |  |  |
| single | 183.65±37.59 | <0.001 | 114.68±69.76 | <0.001 | 105.23±31.06 | <0.001 | 55.48±19.81 | <0.001 |
| Married | 184.6±39.06 |  | 132.43±83.92 |  | 107.38±32.74 |  | 50.69±15.76 |  |
| divorced | 193.43±41.23 |  | 110.34±55.92 |  | 105.99±29.76 |  | 53.5±17.25 |  |
| widow | 181.56±35.88 |  | 136.89±69.53 |  | 113.3±35.56 |  | 52.63±14.43 |  |
| **Socio-economic level** |  |  |  |  |  |  |  |  |
| Low | 186.06±40.3 | 0.06 | 126.58±76.28 | <0.001 | 108.69±33.75 | 0.26 | 51.99±16.09 | <0.001 |
| Middle | 185.6±39.58 |  | 133.78±90.5 |  | 107.51±33.35 |  | 51.3±16.17 |  |
| High | 183.63±37.75 |  | 135.16±80.13 |  | 106.76±31.6 |  | 49.81±15.36 |  |
| **Cigarette smoking** |  |  |  |  |  |  |  |  |
| Yes | 178.97±38.74 | <0.001 | 133.13±84.83 | 0.2 | 104.78±31.55 | <0.001 | 47.5±15.05 | <0.001 |
| No | 189.37±38.16 |  | 130.72±82.86 |  | 115.78±32.33 |  | 52.41±16.12 |  |
| **Alcohol consuming** |  |  |  |  |  |  |  |  |
| Yes | 179.05±40.4 | 0.006 | 146.21±96.89 | 0.03 | 105.2±35.18 | 0.11 | 43.95±11.4 | <0.001 |
| No | 186.66±38.56 |  | 131.05±83.07 |  | 109.2±32.16 |  | 51.22±16.03 |  |
